# Reducing Human-Tsetse Contact Significantly Enhances the Efficacy of Sleeping Sickness Active Screening Campaigns: A Promising Result in the Context of Elimination

**DOI:** 10.1371/journal.pntd.0003727

**Published:** 2015-08-12

**Authors:** Fabrice Courtin, Mamadou Camara, Jean-Baptiste Rayaisse, Moise Kagbadouno, Emilie Dama, Oumou Camara, Ibrahima S. Traoré, Jérémi Rouamba, Moana Peylhard, Martin B. Somda, Mamadou Leno, Mike J. Lehane, Steve J. Torr, Philippe Solano, Vincent Jamonneau, Bruno Bucheton

**Affiliations:** 1 IRD, UMR 177 IRD-CIRAD INTERTRYP, CIRDES 01 BP 454, Bobo-Dioulasso, Burkina Faso; 2 PNLTHA, Ministère de la Santé, Conakry, Republique de Guinee; 3 CIRDES URBIO, 01 BP 454, Bobo-Dioulasso, Burkina Faso; 4 Centre MURAZ, Bobo-Dioulasso, Burkina Faso; 5 Liverpool School of Tropical Medicine, Liverpool, United Kingdom; 6 IRD, UMR 177 IRD-CIRAD INTERTRYP, PNLTHA-Ministère de la Santé, Conakry, Republique de Guinee; Yale School of Public Health, UNITED STATES

## Abstract

**Background:**

Control of gambiense sleeping sickness, a neglected tropical disease targeted for elimination by 2020, relies mainly on mass screening of populations at risk and treatment of cases. This strategy is however challenged by the existence of undetected reservoirs of parasites that contribute to the maintenance of transmission. In this study, performed in the Boffa disease focus of Guinea, we evaluated the value of adding vector control to medical surveys and measured its impact on disease burden.

**Methods:**

The focus was divided into two parts (screen and treat in the western part; screen and treat plus vector control in the eastern part) separated by the Rio Pongo river. Population census and baseline entomological data were collected from the entire focus at the beginning of the study and insecticide impregnated targets were deployed on the eastern bank only. Medical surveys were performed in both areas in 2012 and 2013.

**Findings:**

In the vector control area, there was an 80% decrease in tsetse density, resulting in a significant decrease of human tsetse contacts, and a decrease of disease prevalence (from 0.3% to 0.1%; p=0.01), and an almost nil incidence of new infections (<0.1%). In contrast, incidence was 10 times higher in the area without vector control (>1%, p<0.0001) with a disease prevalence increasing slightly (from 0.5 to 0.7%, p=0.34).

**Interpretation:**

Combining medical and vector control was decisive in reducing *T*. *b*. *gambiense* transmission and in speeding up progress towards elimination. Similar strategies could be applied in other foci.

## Author Summary

Sleeping sickness is a serious neglected tropical disease which has been targeted for elimination by 2020. Currently, control relies on mass screening populations at risk and treatment of cases. This strategy is compromised because it is often impossible to reach more than 75% of the population. An alternate method is to kill the tsetse fly which transmits the parasite causing the disease. To determine the value of adding vector control to medical interventions we undertook a vector control programme alongside a screen and treat programme in the Boffa disease focus of Guinea. We divided the focus into two parts (screen and treat in the western part; screen and treat plus vector control in the eastern part). In the vector control area, there was an 80% decrease in tsetse density and a decrease of disease prevalence from three cases per thousand to one case per thousand with almost no new cases being contracted during the control period. In contrast, incidence was 10 times higher in the area without vector control. Combining medical and vector control was decisive in reducing sleeping sickness transmission and could speed up the elimination process. Similar strategies could be applied in other foci.

## Introduction

Human African Trypanosomiasis (HAT), also known as sleeping sickness, is a lethal parasitic disease that afflicts poor rural communities in remote parts of Africa and is included in the list of neglected tropical disease of the London declaration that are foreseen for elimination as public health problems by 2020 [1]. The disease is caused by trypanosomes transmitted by an insect vector, the tsetse fly. There are two forms of HAT: one, known as gambiense HAT, is endemic in West and Central Africa and causes over 95% of current cases; the other, known as rhodesiense HAT, is endemic in East and southern Africa [2]. The presence of parasites in the brain is associated with a disturbance of the sleep-wake cycle and the progressive appearance of neurological disorders. Eventually, patients fall into a coma and die if not treated. There is no vaccine, and available drugs are toxic and require several days of complete hospitalization despite recent improvements [3].

Active case detection by mass screening of the population and treatment has proven effective in lowering HAT prevalence [2]. However, there are a number of foci where the screen and treat strategy, despite saving many lives and preventing large outbreaks from occurring, has failed to bring HAT under effective control. Low attendance of the population at medical surveys, combined with the existence of human and/or animal carriers with cryptic infections escaping the screen and treat strategy, are important factors impairing the efficacy of control measures targeting confirmed HAT patients only [4]. Whereas vector control is an important arm in the control of animal trypanosomiasis and rhodesiense HAT [5], it has been scarcely used in gambiense HAT in part because vector control was considered too costly and too difficult to deliver in resource poor settings [6]. Recently, a new cost effective and environment friendly technology, based on the deployment of small insecticide-impregnated targets has been developed [7,8] and was for the first time implemented here in an active gambiense HAT focus. Our goals were to evaluate its efficiency (i) in lowering tsetse densities and human tsetse contact and (ii) in reducing the burden of HAT.

The study was performed in the mangrove area of Boffa, one of the most active foci in West Africa. Mangroves display a favorable biotope for *Glossina* (*G*.) *palpalis* (*p*.) *gambiensis* leading to a high exposure of the population to tsetse bites. The population living in mangrove areas is often difficult to access and characterized by an important daily and seasonal mobility. Both are major factors impairing the efficacy of medical surveys and this is in part responsible for the persistence of HAT in such foci. As part of a strong resolve by the government of Guinea to tackle HAT in the coastal part of the country, a vector control intervention was combined with the classical medical one, in order to improve HAT control and to accurately measure the added value of integrating vector interventions in the country’s control strategy. The focus was divided into two areas separated by the Rio Pongo River. Medical surveys covering the entire population were performed in 2012 and 2013 but insecticide impregnated targets were only deployed on the eastern bank. We demonstrate a remarkable decrease of *T*. *b*. *gambiense* transmission in the area where the two strategies were combined.

## Methods

### Ethical statement

This study is part of a HAT elimination programmer in coastal Guinea (ElimTrypGui) that was approved by the Guinean Minister of Health (234/MSHP/CAB/2013). The Ministry of Health of the Republic of Guinea has approved the study protocols and given the administrative authorizations for the activities performed within this study by the National Control Programme. Mass screening and treatment of HAT patients were performed according to the national Guinean HAT procedures as recommended by the World Health Organization. Information on HAT and the study objectives was provided to the population both (i) on a household basis during the census of the population and distribution of invitations to participate in medical surveys and (ii) through discussion groups organized with the Boffa district health authorities and village administrative and religious authorities. Consent to participate was oral and reflected by the participation or not in the medical surveys. No human biological samples other than those required for HAT diagnosis were taken from participants in the frame of this study.

### Study area and control strategies

**Fig 1:**
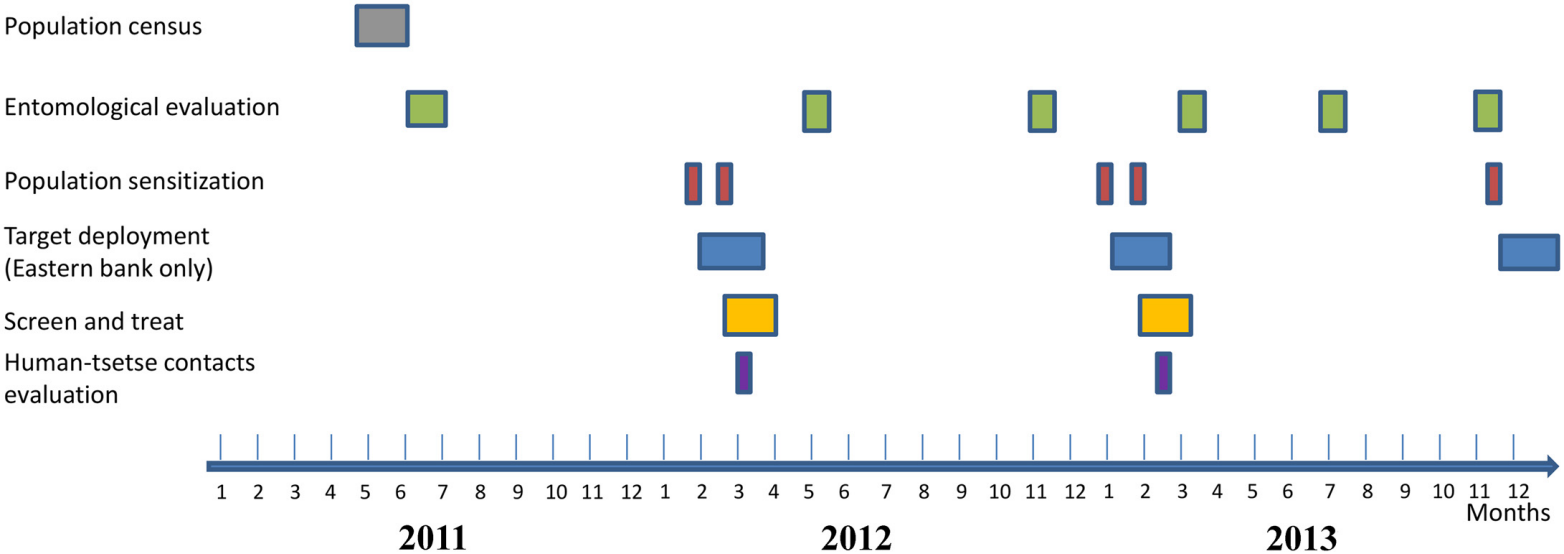
Flow chart showing the different activities implemented in the sleeping sickness focus of Boffa.

The study was carried out in the Boffa HAT focus located in a mangrove area of coastal Guinea. Most of the population is from the Soussou ethnic group and lives in small villages scattered along mangrove channels. The main occupations are rice cultivation, fishing, wood cutting and salt extracting, all activities that bring the population into close contact with *G*. *p*. *gambiensis* the only vector of *T*. *b*. *gambiense* in these areas. Population characteristics, human settlements and density and geographic distribution of tsetse flies in this focus have already been described in detail [9]. The focus was divided into two parts separated by the Rio Pongo River. In “Boffa West” (western bank, 320 km^2^) only the medical activities (“screen and treat”) were performed. In “Boffa East” (320 km^2^) insecticide-impregnated targets were deployed to reduce tsetse densities in addition to medical surveys. A detailed flow chart of activities is given in Fig 1.

HAT has been known to occur in Boffa since colonial times. Prior to this study, efforts to control the disease in this focus has been through passive detection of HAT patients at the Dubreka treatment center (located 150 km away) and targeted screening of villages reporting cases by mobile teams. A total of 229 patients from the Boffa focus were diagnosed and treated by the NCP between 2002 and 2009.

### Population census and medical surveys

**Fig 2:**
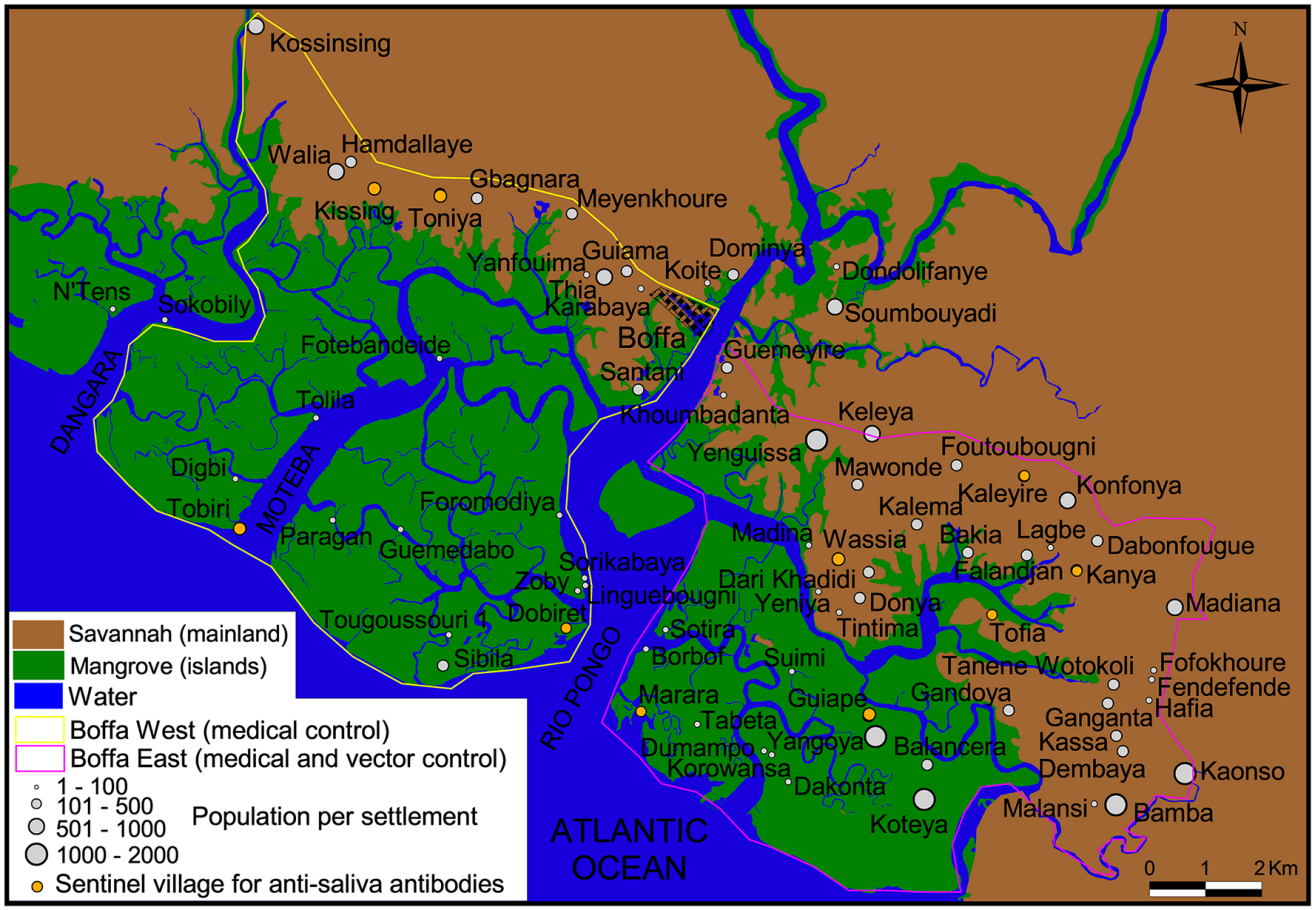
Map of the study area indicating the location of settlements.

An exhaustive census of the population was performed in May 2011 during which each household was geo-referenced. For each inhabitant, first name, second name, sex and age were recorded. Each household family was given a unique code, to which a number was added for each family member thus giving a unique individual identifier. The 74 human settlements (villages, hamlets, encampments) that were mapped in the study area are shown in Fig 2. They comprised a population of 20,496 inhabitants: 29 settlements and 4,474 inhabitants in Boffa West, and 45 settlements and 16,022 inhabitants in Boffa East, respectively. Newly settled families and seasonal workers presenting to the medical surveys in 2012 (n = 4,094) and 2013 (n = 3,189) were also added to the census database which finally included 27,779 individuals in 2013.

Two medical surveys, in which all villages from the entire study area were visited by a mobile medical team, were organized in 2012 just after target deployment. This was repeated in 2013. Prior to each survey, population sensitization was undertaken to inform village authorities of the passage of the medical team and to distribute household census printed sheets that were used during surveys to record attendance and results of diagnostic tests. During surveys, all participating inhabitants were tested with the Card Agglutination Test for Trypanosomiasis (CATT) [10]. Two-fold plasma dilutions were tested for each positive individual and parasitological tests (direct microscopic examination of lymph node aspirate and/or mini-anion exchange centrifugation test on buffy-coat [11]) were performed for all individuals with CATT end titer > 1/4. Since the CATT is known to be prone to unspecific agglutinations, one ml of left over plasma was sampled from every CATT positive subjects and was frozen until use. These samples were used to perform the highly specific immune trypanolysis test (TL) using cloned populations of *T*. *b*. *gambiense* variable antigen type (VAT) LiTat 1.3 [12,13]. Only individuals that tested positive to the CATT and to TL were considered to harbor *T*. *b*. *gambiense* specific antibodies. At the end of each medical survey, all parasitological positive subjects were treated according to the national treatment procedure, which is pentamidine for 1^st^ stage infections, and the Nifurtimox-Eflornithine combination therapy for 2^nd^ stage infections, as recently recommended [3].

### Vector control and monitoring

#### Entomological monitoring

Baseline entomological data (the reference data-sampling round 0) were collected from the study area prior to the intervention in May 2011 [9] and were used to direct target deployment in the vector control area. Throughout the entire programmer five monitoring surveys (rounds 1 to 5) were implemented to assess changes in the tsetse density in the study area. For this, 58 geo-referenced sentinel traps were positioned for 48 hours at fixed sites to allow comparison between surveys: 47 were located in Boffa East (vector control) and 11 in Boffa West (no vector control).

#### Target deployment

The aim of target deployment was not to eliminate tsetse flies but rather to reduce human-tsetse contact in order to protect people from infective tsetse bites. Targets were thus deployed in places where people live and work (favorable vegetation around villages, pirogue jetties, watering points, agricultural sites, main foot paths and mangrove channels). The targets have a central panel (0.5 x 0.5 m) of blue polyester impregnated each with deltamethrin (300 mg/m^2^) flanked by two panels of black polyethylene netting (0.5 m high x 0.25 m wide each) also insecticide impregnated. Each target also included a bag containing 2 ml of octenol and para-cresol (1:2) as olfactory attractant for tsetse. Targets were bought from a commercial company every year, and were erected using locally cut wooden sticks or were hung from the branches of trees (see S1 Text for more details). In February 2012, a total of 3,926 insecticide-treated targets were deployed in Boffa East. Targets were replaced by new ones each year at the end of the rainy season in december 2012 and december 2013. Additional targets were deployed at new sites to improve spatial coverage, giving a total of 4,673 geo-referenced sites used for targets deployment and a mean number of 15 targets/km^2^ (S1 Fig).

#### Evaluation of human exposure to tsetse flies

In order to measure the level of human exposure to tsetse bites, the IgGs responses directed against the Tsgf1_18-43_ synthetic peptide shown to be specific to tsetse saliva [14–16] were quantified. Ten sentinel villages were selected for this purpose (four in Boffa West and six in Boffa East, Fig 2). In these villages plasma samples were collected from heparinised capillary tubes taken from all inhabitants older than five years old who participated in medical surveys. In 2012, 252 and 652 samples were obtained from Boffa West and Boffa East, respectively. In 2013, 395 and 539 were obtained from Boffa West and Boffa East, respectively. The quantification of anti-Tsgf1_18-43_ IgGs was performed by an indirect-ELISA assay as described previously [16].

### Statistical analyses

The tsetse densities obtained at every monitoring period in both the vector control area and the non-vector control area were compared using the Wilcoxon matched-pairs signed-rank test using T0 data as the reference. The impact of the vector control intervention on the evolution of tsetse densities was evaluated by constructing a mixed linear regression model of the mean number of catches/day/trap including the trapping site as a random effect and the period of sampling and deployment of impregnated targets as fixed effects. The Wilcoxon matched-pairs signed-rank test was used to compare the evolution of Tsgf1_18-43_ IgG responses (2012–2013) in sentinel villages from Boffa West and Boffa East. The chi square test was used to assess prevalence differences of specific serology and HAT between years or study areas. The Fisher exact test was used to compare incidences of specific sero-conversion events and of confirmed HAT cases.

## Results

### Vector control

#### Impact on tsetse densities

**Fig 3:**
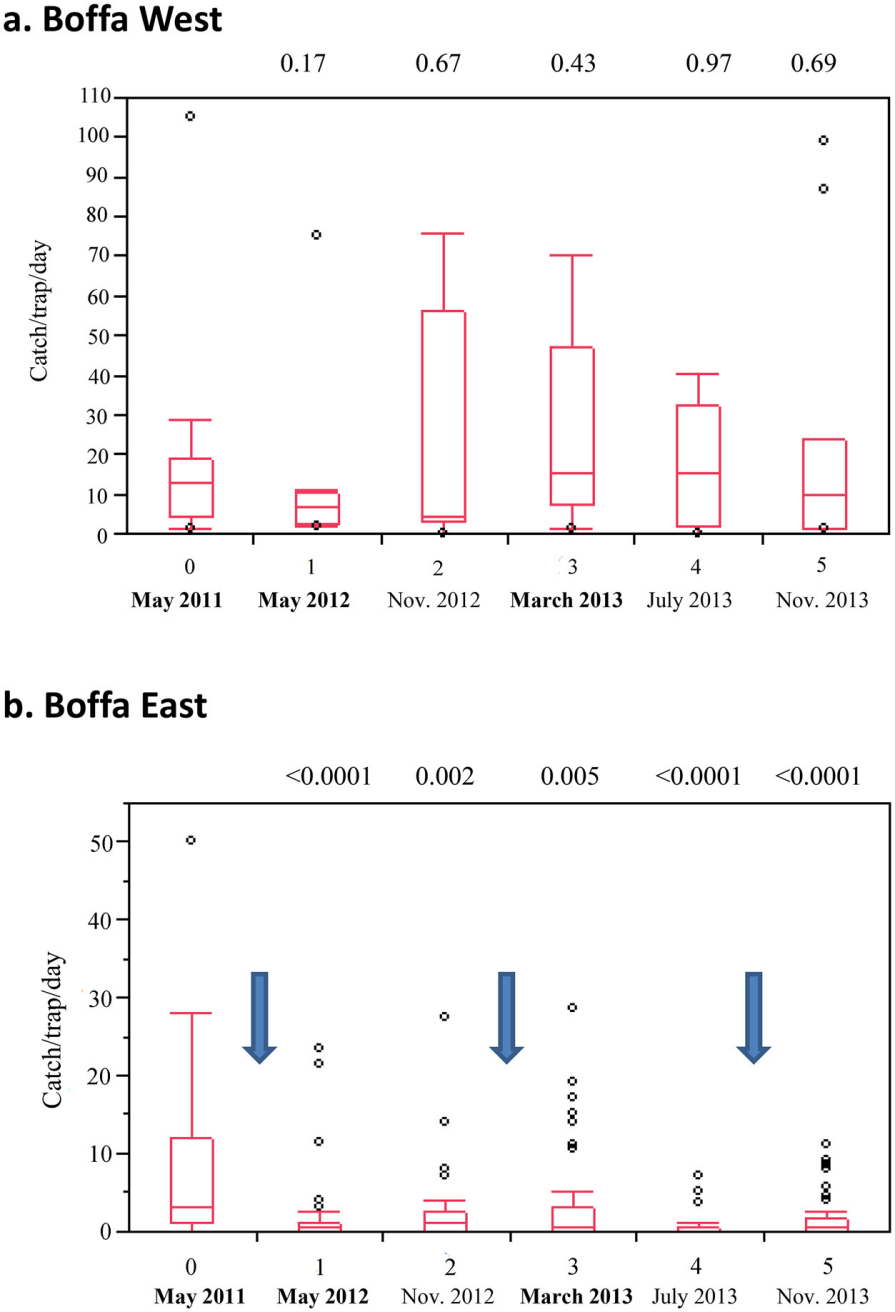
Evolution of tsetse densities during the study period in the Boffa focus. Box plots showing the median and 10^th^, 25^th^, 75^th^ and 90^th^ centiles of catches of tsetse from six sampling rounds (May 2011—November 2013) in Boffa West (a.) where no tsetse control intervention where initiated and Boffa East (b.) where insecticide-impregnated targets were deployed. Blue arrows indicate insecticide-impregnated target deployment/replacement. P-values of the difference between each monitoring round and pre-treatment data in May 2011 are indicated (Wilcoxon matched-pairs signed-rank test).

The evolution of tsetse densities in the study area, as measured by sentinel traps, is shown in Fig 3. In the area where insecticide-impregnated targets were deployed, the number of tsetse caught was found to be significantly reduced at all-time points as compared to the pre-treatment evaluation. Starting from a median of 3 flies/trap/day in the pre-intervention monitoring round, tsetse densities decreased to 0.5 one year and half later (round 5), corresponding to an overall 80% decrease. In contrast, no significant differences were observed at any of the monitoring rounds, in the area where no vector control was initiated (Boffa West). This study was not designed to demonstrate seasonal effects, as most monitoring rounds were performed during the dry season (except the one in July 2013). In mangroves, environmental conditions are favorable to tsetse flies all year long, it is nevertheless known that the efficacy of tsetse catches are affected by factors such as wind, rain or tide coefficients. To take this into account, we used a mixed linear model including the tsetse traps as a random effect and the date of sampling and deployment of targets as fixed effects (Table 1). We indeed detected a significant effect of the date of sampling (p = 0.0027)) and a highly significant effect of target deployment (p<0.0001) on tsetse densities.

**Table 1:** Mixed linear regression model of the impact of target deployment on tsetse catches in the Boffa focus.

Fixed effects	Nb of coefficient		Means of catches/day Least squares (± standard error)	F ratio	Prob. >F
**Sampling round**	5		**-**	3.7459	0.0027
**Target deployment**	1	yes	2.99 (± 1.29)	26.7620	< 0.0001
		no	18.17 (± 2.63)		

Mixed linear regression was used to model the response variable that was the mean number of catches/day/trap. The trapping site was included as a random effect and the period of sampling and deployment of impregnated targets as fixed effects.

#### Evolution of human exposure to tsetse flies

**Fig 4:**
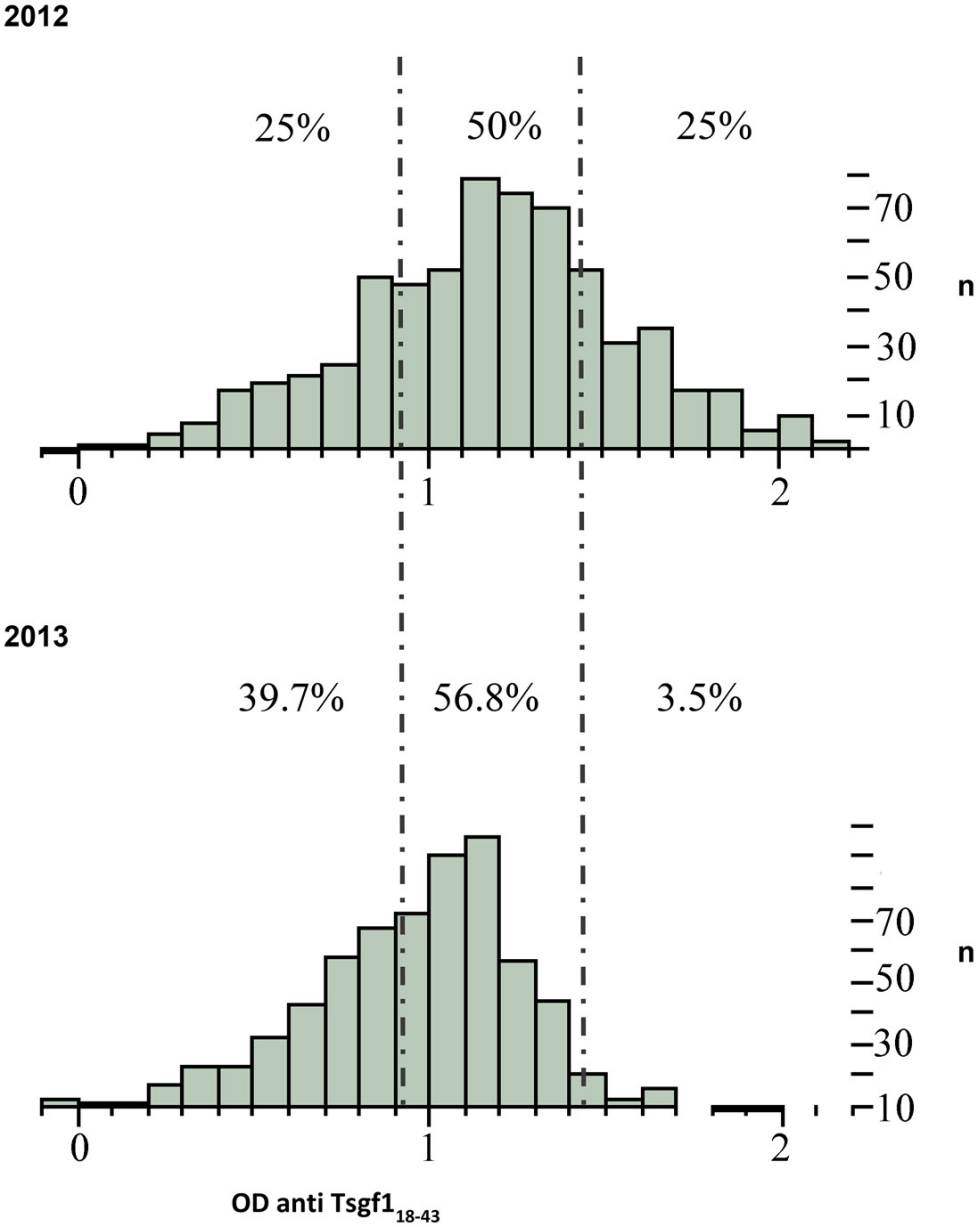
Histogram showing the changes of anti Tsgf18-43 IgGs responses in 2012 and 2013 in the vector control area (Boffa East). Distributions of anti Tsgf_18-43_ Elisa optical densities obtained from 652 individuals in 2012 and 539 individuals in 2013 sampled in sentinel villages of the vector control area (Boffa East). Dashed lines represent the quartiles of the IgG response calculated in 2012.

In agreement with the entomological data, a highly significant decrease of Tsgf1_18-43_ ELISA titres was observed in sentinel villages from the vector control area (p<0.0001) in 2013. In contrast those from the area without vector control displayed a modest increase (p = 0.03). Analysis of the distribution of anti- Tsgf1_18-43_ IgG levels in the vector control area in 2012 and 2013 suggest that a major impact of target deployment was to reduce the number of high responders that are likely to be the most exposed to tsetse bites. Whereas individuals with high Tsgf1_18-43_ ELISA titres (optical density >1.42) represented 25% of the total population in 2012, this proportion fell to less than 4% in 2013 (Fig 4).

### Disease prevalence and incidence

Out of the 27,779 individuals registered in the census database in 2013, 17,203 participated in at least one of the two surveys performed in 2012 and 2013 (61.9%) and 10,576 participated in neither (38.1%). Population coverage over the two surveys was higher in Boffa West (73%) than in Boffa East (58.4%).

During the 2012 survey, *T*. *b*. *gambiense* specific antibodies were detected in 0.93 and 0.43% of the population in Boffa West and Boffa East, respectively, whereas the presence of trypanosomes was confirmed by microscopy in 0.54 and 0.30%. In contrast to the situation in Boffa West where the prevalence of specific serology and HAT remained stable between 2012 and 2013, a significant decrease of both specific serology (0.2% in 2013; p = 0.01) and HAT prevalence (0.11% in 2013; p = 0.01) was observed in Boffa East where impregnated targets were deployed (Table 2).

**Table 2:** Evolution of seroprevalence and disease prevalence in the Boffa HAT focus.

	Boffa West (no vector control)			Boffa East (vector control)		
	2012	2013	P^4^	2012	2013	P^4^
Number tested	3344	2885		7927	6564	
CATT^1^ positive	103 (3.08%)	68 (2.36%)	0.08	134 (1.69%)	87 (1.32%)	0.07
Specific serology (CATT+/TL+)^2^	31 (0.93%)	26 (0.9%)	0.92	**34 (0.43%)**	**13 (0.2%)**	**0.01**
Confirmed HAT^3^	18 (0.54%)	21 (0.73%)	0.34	**24 (0.30%)**	**7 (0.11%)**	**0.01**
Unconfirmed with specific serology	13 (0.39%)	5 (0.17%)	0.11	10 (0.13%)	6 (0.09%)	0.53

Prevalence data (in brackets) were calculated according to the total population tested in each of the two areas in 2012 and 2013. Former HAT patients who were diagnosed and treated prior to the 2012 survey (14 and 11 in Boffa West and Boffa East respectively) and to the 2013 survey (26 and 28 in Boffa West and Boffa East respectively) were removed from this analysis.

^1^ Card Agglutination Test for Trypanosomiasis

^2^ Individuals that were both positive to the CATT and the *T*. *b*. *gambiense* immune trypanolysis test were considered to harbour a specific serology to *T*. *b*. *gambiense*.

^3^ Trypanosomes were detected by direct microscopic examination of lymph node aspirates or by the mini-anion exchange centrifugation test performed on buffy coat.

^4^ Pearson’s Chi-squared test P-value (2012–2013 comparison).

Similar observations were made on the population of new comers (not present during the population census in 2011 but who participated in medical surveys in 2012 and 2013). In 2012, prevalence of HAT in this population was 0.2% (7/2806) and 0.3% (4/1288) on the Eastern and Western Bank, respectively. In contrast no HAT cases were detected in 2094 newly settled inhabitants in Boffa East in 2013 whereas HAT prevalence remained at 0.3% (3/1095) in Boffa West. In order to accurately assess the impact of HAT control on *T*. *b*. *gambiense* transmission we then calculated the number of specific seroconversion events amongst individuals who tested negative to the CATT in 2012 and who were seen again in 2013 (1,279 in Boffa West and 2,277 in Boffa East). In Boffa West, 16 specific sero-conversion events were detected and 14 were confirmed parasitologically (incidence of 1.09%). The incidence of new seroconversion events was significantly lower in Boffa East (incidence = 0.07%; p<0.0001) where only two new HAT cases were diagnosed (Table 3).

**Table 3:** Incidence of new infection events.

	Boffa West no vector control		Boffa East with impregnated targets		
	n	Incidence (%)	n	Incidence (%)	P^3^
CATT negative in 2012	1279		2777		
CATT+ TL+ in 2013^1^	**16**	**1.25**	**2**	**0.07**	**<0.0001**
Confirmed HAT in 2013^2^	**14**	**1.09**	**2**	**0.07**	**<0.0001**

The incidence of new specific sero-conversion events and of confirmed HAT cases was calculated in Boffa West and Boffa East based on individuals that tested negative to the CATT in 2012 and who were seen again during the 2013 medical survey.

^1^ Positive to the CATT test and positive to the *T*. *b*. *gambiense* immune trypanolysis test

^2^ Trypanosomes were detected by direct microscopic examination of lymph node aspirates or by the mini-anion exchange centrifugation test performed on buffy coat.

^3^ P-value (Fisher exact test).

## Discussion

Our results show that the deployment of targets in the eastern part of the Boffa focus had a very significant impact on HAT prevalence and incidence. Although the decrease in tsetse density was not greater than 80%, this was enough to significantly reduce *T*. *b*. *gambiense* transmission within a few months, whereas in the “no vector control” area, the number of HAT cases remained stable. The need to add vector control activities to medical activities for gambiense HAT has already been pointed out [6,17,18], but the present study is the first one to accurately measure what it brings in terms of reduction of HAT transmission.

### The limits of the screen and treat strategy

Up to now, HAT has relied heavily on the screen and treat strategy where at risk populations are visited by mobile medical teams, using the CATT as the mass screening test and treating microscopically confirmed HAT patients. Although this strategy has proven very efficient in lowering disease prevalence and has saved thousands lives, it may not be sufficient when the objective is the elimination of the disease from a focus. The existence of human asymptomatic carriers in Guinea has been reported [19] and is also suggested here by the detection of a number of individuals displaying *T*. *b*. *gambiense* specific antibodies but negative parasitological results. It is noteworthy that in Boffa West where only the screen and treat strategy was applied, we did not observe any significant decrease in HAT prevalence between 2012 and 2013 despite a population coverage estimated to be around 70%. Instead, a high incidence of new infection events (>1%) was observed in the population who tested CATT negative in 2012. This illustrates that a sufficient number of infected hosts remained in the area at the end of the 2012 survey to infect tsetse flies. In this context, a high number of costly screening rounds would probably be required to bring HAT under effective control. The fact that population coverage tends to decrease following repeated medical surveys (due to population fatigue) is a known phenomenon reported from a number of countries including the Democratic Republic of Congo [20] and should be taken into account in planning HAT elimination strategies.

### Adding vector control significantly increases the efficacy of medical surveys to lower transmission levels

In sharp contrast to the situation described above and despite lower population coverage, HAT prevalence was reduced by a magnitude of three (from 0.3 to 0.1%) on the Eastern bank of the focus where insecticide-impregnated targets were deployed. Furthermore, the fact that only two new infection events were detected amongst 2,777 individuals testing negative to the CATT in 2012 (incidence = 0.007) indicates that *T*.*b*. *gambiense* transmission had fallen to very low levels in this area. More importantly, this sharp effect was associated with a reduction of tsetse densities of only 80%, indicating that partial vector control, combined with active screening, significantly impacts on parasite transmission and speeds up the elimination process. Because mangroves represent probably one of the most challenging environments for vector control as large areas are not accessible, impregnated targets were mainly deployed to limit exposure in areas where the population is highly exposed to tsetse bites and thus to decrease the risk of being infected or to infect tsetse flies. Analysis of the antibody response to a tsetse saliva specific epitope (Tsgf) in the population of sentinel villages from the vector control area accordingly demonstrated a significant decrease of antibody titers just one year after initial target deployment. Although an important proportion of inhabitants still exhibited intermediate antibody titers in 2013 indicating that they were probably still exposed to tsetse flies, the proportion of high responders dropped sharply from 25% to less than 4% suggesting that intense exposure to tsetse bites had been strongly reduced in the population. A high level of human tsetse contacts thus appears crucial for *T*. *b*. *gambiense* transmission, a condition that would appear to have been reduced by the deployment of insecticide-impregnated targets on the eastern bank of the Boffa focus.

Targeting areas of human tsetse contacts to reduce the level of human exposure to tsetse flies thus appear an efficient tool to use in combination with the screen and treat strategy, especially where human populations are highly exposed to tsetse flies. During this two year intervention, 12,000 impregnated targets were used. Compared to the total population living in the area, this represents less than a target/inhabitant. In addition, targets are easy to set-up and deployment in areas of human activities could be transferred to the local communities with minimum intervention from the central level in order to secure vector control sustainability. How long such measures should be maintained in a given focus remains an open question but will critically depend on the existence of other nearby foci from where infected hosts could migrate. Such approaches combining vector and medical control could be applied in other biotopes where they could prove even more effective. The next place where such vector control operation will be undertaken in addition to the medical approach is of course Boffa West.

## Supporting Information

S1 Fig Map showing the distribution of insecticide impregnated targets deployed in the Boffa focus. (TIF)

S1 Text Illustrated description of target deployment (A-) and entomological monitoring of tsetse densities (B-) in the Boffa HAT focus. (DOCX)
