# Supplementary figures and images for: Reducing Human-Tsetse Contact Significantly Enhances the Efficacy of Sleeping Sickness Active Screening Campaigns: A Promising Result in the Context of Elimination
Source: PLoS Negl Trop Dis. 2015 Aug 12;9(8):e0003727. doi: 10.1371/journal.pntd.0003727 (PMC4534387; doi:10.1371/journal.pntd.0003727)

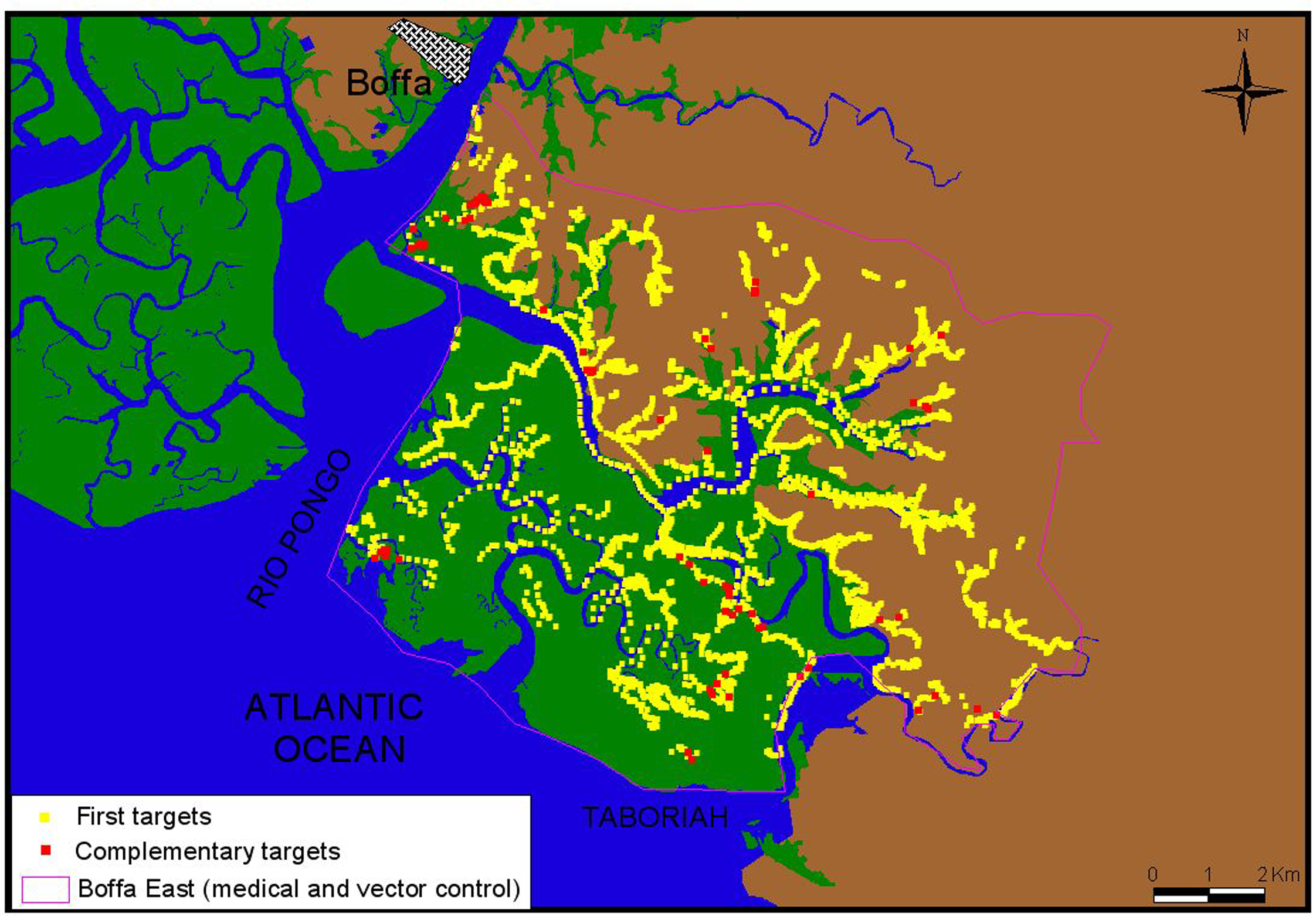

Supplement: S1 Fig — (TIF) [file pntd.0003727.s001.tif]
