# Supplementary material for: Reducing Human-Tsetse Contact Significantly Enhances the Efficacy of Sleeping Sickness Active Screening Campaigns: A Promising Result in the Context of Elimination
Source: PLoS Negl Trop Dis. 2015 Aug 12;9(8):e0003727. doi: 10.1371/journal.pntd.0003727 (PMC4534387; doi:10.1371/journal.pntd.0003727)

## A- Target deployment

### Objectives

The purpose of this campaign was not to eradicate tsetse flies on the Eastern Bank of the Rio Pongo river, but to reduce contact between flies and humans in order to reduce HAT incidence. For this reason and because many areas of the mangrove are completely inaccessible, targets were mainly deployed in areas of intense human activities.

These are as follows:

1. mainland :
   1. Housing (towns, villages, settlements, encampments)
   2. Area frequented by humans
      1. forest gallery: bridges, water supply points, fishing sites
      2. shallows: Paddy fields, gardens, brick fabrication sites
      3. interfluves: sorghum, cassava, palm trees (oil extraction), orchards (orange, kola), charcoal extraction
      4. interface savanna- mangrove: ports, jetties (landing stages), net or basket fishing points, natural sources
   3. Other sites preserved by humans
2. Mangrove (continental and mangrove islands)
   1. Islands with exactly the same types of subdivisions as in point 1b above.
   2. Mangrove tree islands
      1. villages, settlements, encampments, landing stages
      2. paddy fields, salt extraction
      3. fishing sites, wood exploitation, oyster collection sites (plates 1 a and b)
      4. mangrove channels, mainly narrow and medium ones.

Plate 1: Women coming back with shellfish (A) and wood (B)


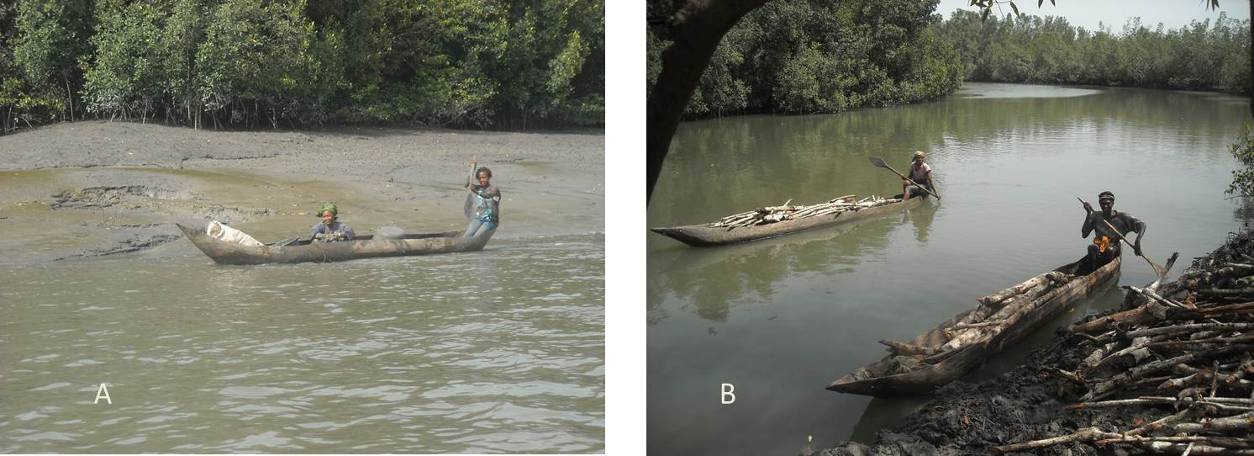


### Height above the ground

Targets were deployed using either wooden sticks cut *in situ* or by directly hanging them on the branches of trees at adapted suitable height. This height mainly depended on the area or the site where the target was set. In flat areas on the mainland, the distance between the bottom edge of the target and the ground was between 15 and 30 cm (plate 2). This is the normal height at which any target should be put, since it corresponds to the normal height of the flight of this tsetse species. However, when a target was threatened to be hidden by the vegetation or to be flooded by the tide, the distance above the ground was increased accordingly.

Plate 2: A target deployed in a “normal” way on the bank of a small river


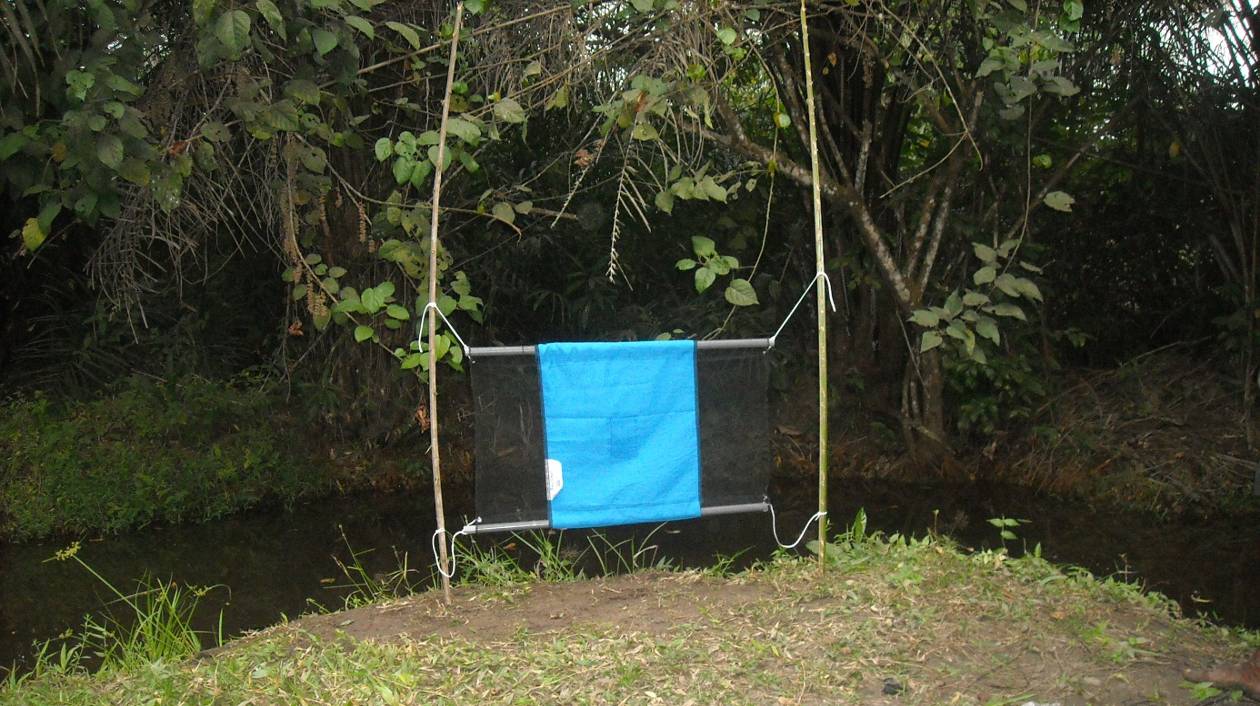


Within the mangrove, all the targets were set according to the water level at the highest tides(Plate 3).

Plate 3: Deployment of a target in a channel at low tide


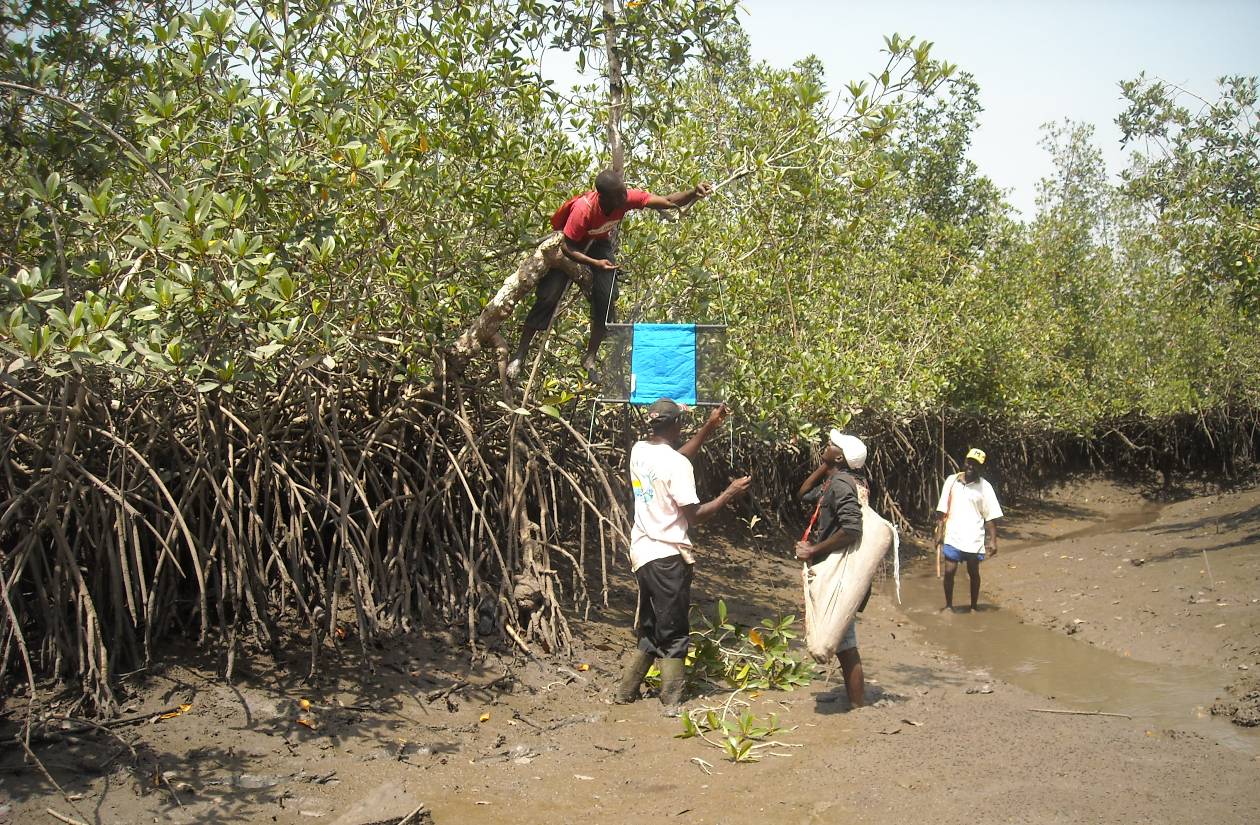


### Distance between targets

The density of targets by unit of surface within an area depends on the epidemiological importance of this area. On areas like water sources, wells, jetties where human presence is permanent, targets are deployed wherever we think it is necessary, and to present targets to cover the different angles of tsetse approach to reduce the contact with tsetse. At other sites, where access is much more difficult, “preventive” targets were deployed. In such situation, distance between two consecutive targets may go up from the normal spacing of 50 meters to 100 -150 meters.

As said previously, the deployment was concentrated on critical areas where the probability of contact between human and tsetse is high. For example, jetties (Plate 4 a) are used as departure or arrival points to/from fields, fishing sites, salt extraction or cutting wood while rivers (Plate 4b) are used either for bath or for washing.

Plate 4: Targets deployed at a jetty (A) and at a washing point


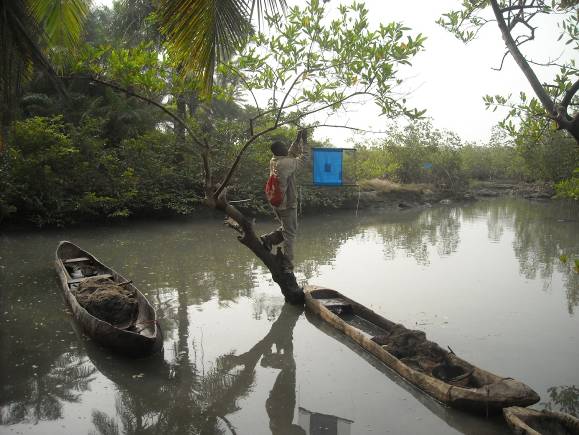


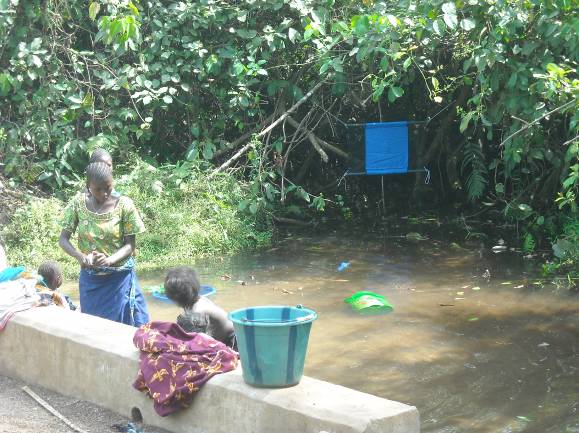


B- Monitoring tsetse densities

During the survey on the islands and particularly in mangrove, work was mainly done by motor boat (Plate 1), hired in Boffa. On the mainland sites were generally accessed by motor bike or on foot.

Plate 1: Deployment of a floating trap


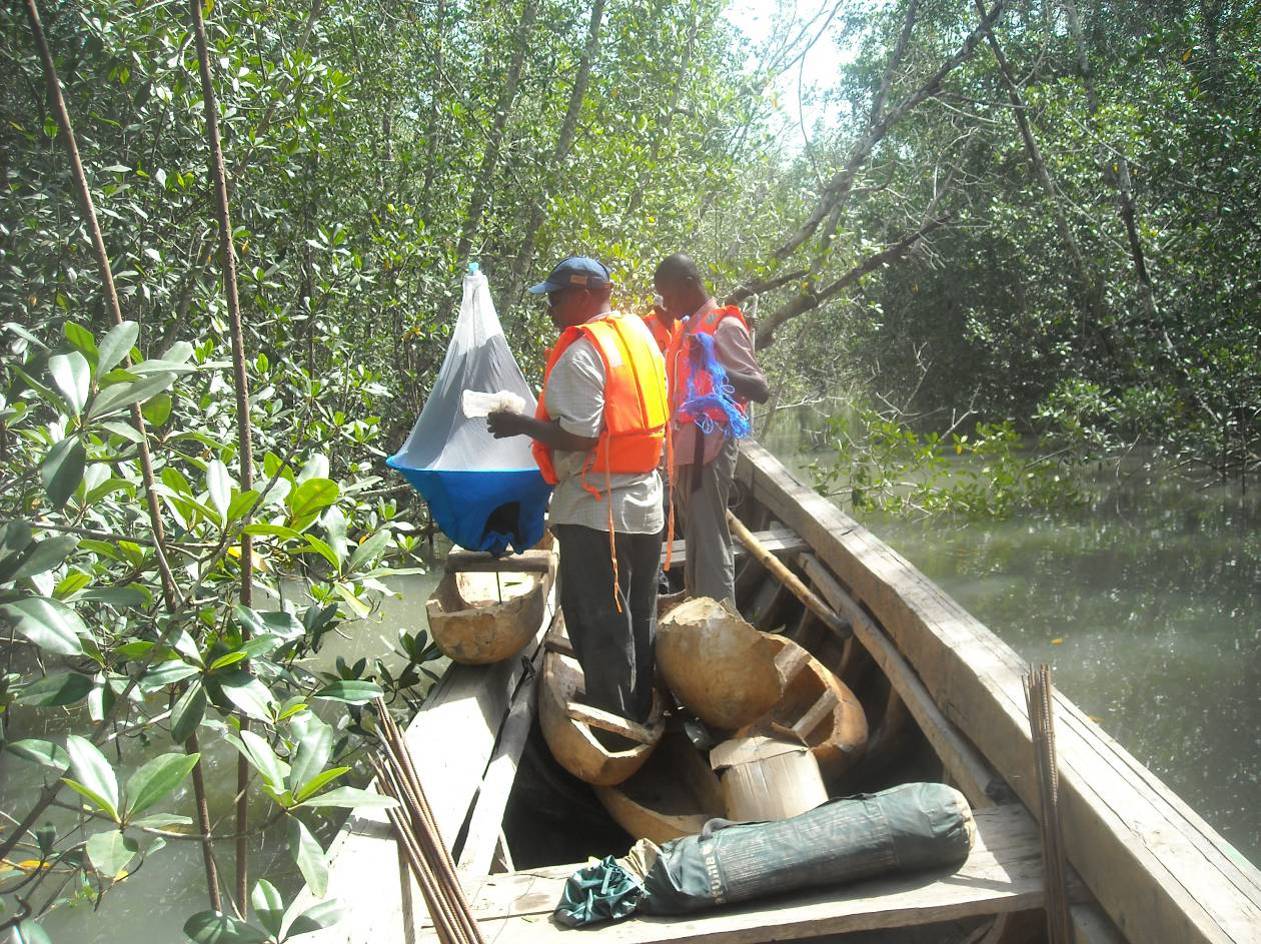


Biconical traps were used for surveys. On the mainland, the standard one was used but on the different mangrove channels, we used either floating traps (Laveissière et al., 2011)^1^ (plates 1 a & b) or suspended traps (plates 2 a & b). Traps were set up for 48 hours but the cages were collected every day when it was logistically possible.

Plate 2: Floating trap at high (A) and low (B) tides


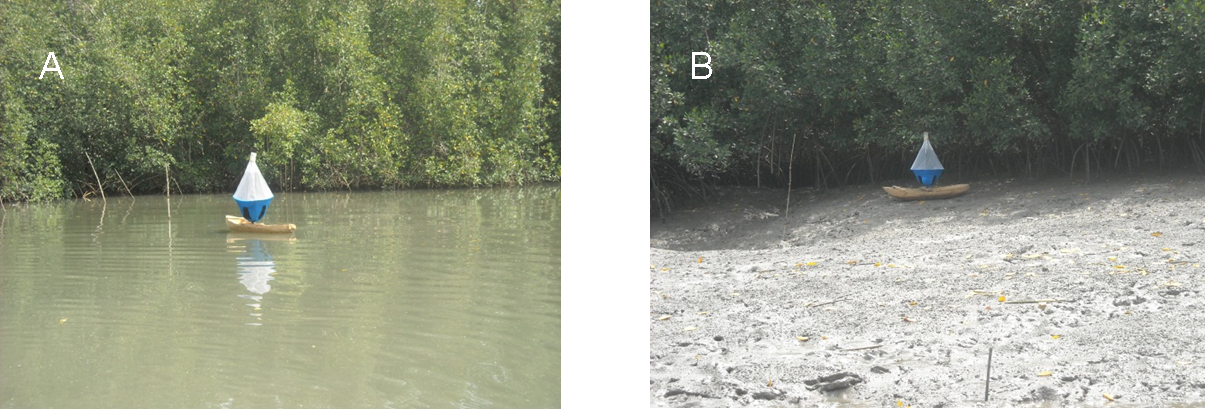


Plate 3: Pending trap at high (A) and low (A) tides


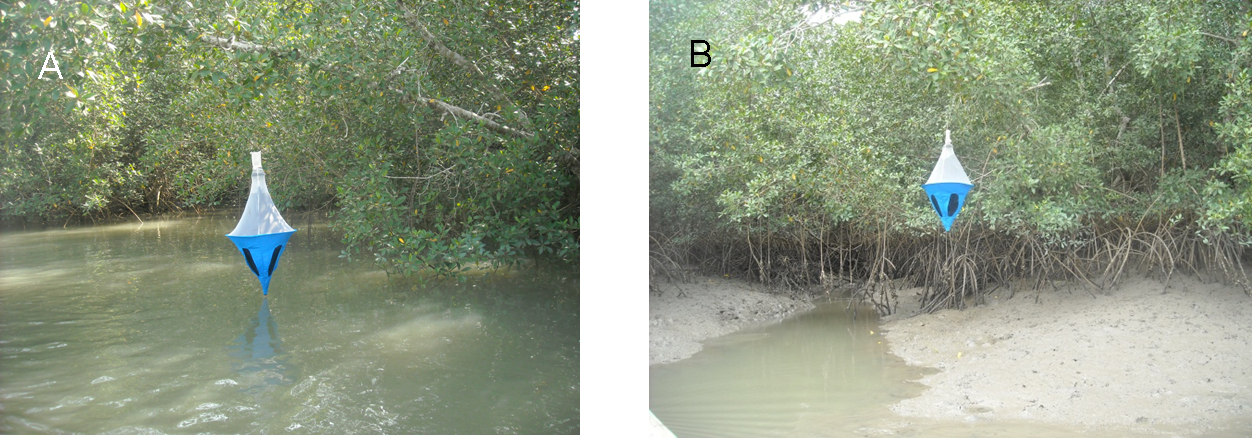

Supplement: S1 Text — (DOCX) [file pntd.0003727.s002.docx]
